# Natural evolution of perforating wounds of 30% extension of the left diaphragm and the anatomopathological characteristics of its healing. Experimental Study

**DOI:** 10.1590/0100-6991e-20223162-en

**Published:** 2022-06-23

**Authors:** THIAGO SOUZA LA-FALCE, DINO MARTINI, MARCIO BOTTER, ROBERTO SAAD

**Affiliations:** 1 - Faculdade de Ciências Médicas da Santa Casa de São Paulo, Departamento de Cirurgia - São Paulo - SP - Brasil; 2 - Faculdade de Ciências Médicas da Santa Casa de São Paulo, Departamento de Ciências Patológicas - São Paulo - SP - Brasil

**Keywords:** Diaphragm, Injuries, Wound Healing, Hernia, Diaphragmatic, Traumatic, Diafragma, Ferimentos e Lesões, Cicatrização, Hérnia Diafragmática Traumática

## Abstract

**Introduction:**

diaphragmatic injury is a challenge for surgeons. It is an injury that can be isolated. It is frequent in penetrating thoracoabdominal trauma. It represents a diagnostic challenge and the ideal approach is not yet well established. The occurrence of spontaneous healing of these injuries is still much discussed and even more, if it does, what is the healing mechanism?

**Objective:**

to macroscopically and histologically evaluate the natural evolution of perforation and cutting wounds equivalent to 30% of the left diaphragm.

**Method:**

50 specimens of rats underwent a surgical procedure and, after 30 days, were euthanized and those that presented scar tissue in the diaphragm, the samples were submitted to histopathological study, using the hematoxylin and eosin stains, Massons trichrome and Picrosirius to assess the presence of collagen or muscle fibers (hyperplasia) in the scar.

**Results:**

it was found that healing occurred in diaphragmatic injuries in 90% of rats. We also observed the presence of fibrosis in all analyzed samples.

**Conclusion:**

Spontaneous healing occurred in most diaphragmatic injuries and the inflammatory reaction represented by the presence of fibrosis and collagen deposition was observed in all our samples. Muscle fiber hyperplasia did not occur.

## RESUMO

**Introdução:**

o ferimento diafragmático é um desafio para os cirurgiões. É uma lesão que pode ser isolada. É frequente nos traumas penetrantes toracoabdominais. Representa um desafio diagnóstico e a conduta ideal ainda não está bem estabelecida. A ocorrência da cicatrização espontânea dessas lesões é ainda muito discutida e mais ainda, se ocorre, qual o mecanismo de cicatrização? Objetivo: avaliar macroscopicamente e histologicamente a evolução natural das feridas perfuro cortantes equivalentes a 30% do diafragma esquerdo.

**Método:**

50 espécimes de ratos, foram submetidos a procedimento cirúrgico e, após 30 dias, foram submetidos à eutanásia e aqueles que apresentaram tecido cicatricial no diafragma, as amostras foram submetidas a estudo histopatológico, usando as colorações de hematoxilina e eosina, tricrômico de Masson e Picrossirius para avaliar a presença de colágeno ou de fibras musculares (hiperplasia) na cicatriz.

**Resultados:**

verificou-se que ocorreu nas lesões diafragmáticas, a cicatrização em 90% dos ratos. Observamos também, a presença de fibrose em todas as amostras analisadas.

**Conclusão:**

ocorreu cicatrização espontânea na maioria das lesões diafragmáticas e a reação inflamatória representada pela presença de fibrose e deposição de colágeno foi observada em todas as nossas amostras. Não ocorreu hiperplasia de fibras musculares.

## INTRODUCTION

Injuries in the so-called “thoracoabdominal transition zone” are the main cause of diaphragmatic injury^1^ ^-^ ^4^.

The limits of this region were described by Madden^5^ in 1989: fourth intercostal space anteriorly, tip of the scapula posteriorly, sixth intercostal space laterally, and the epigastrium inferiorly.

Most diaphragmatic injuries are promptly diagnosed and treated. According to Bernini^6^, these lesions are smaller than 2cm in 76% of the cases, which is also the understanding of other authors^7^ ^,^ ^8^.

However, there is a group of patients who suffer injuries in this area, either on the right or on the left, resulting in diaphragmatic lesions that are difficult to diagnose. These are injuries only to the diaphragmatic muscle, without involvement of other viscera^9^ ^,^ ^10^, which may go unnoticed^11^ ^,^ ^12^. How will these injuries evolve? Will diaphragmatic hernia always ensue, or will the lesion progress into spontaneous healing?

Carter and Giuseffi^13^ stated that diaphragm wounds are prone to not healing due to the migration of viscera driven by the thoracoabdominal pressure gradient. The evolution to herniation of abdominal contents into the pleural cavity and its potential consequences of incarceration and strangulation may occur after a variable period of many years^14^ ^,^ ^15^.

The natural history of isolated diaphragmatic injury is not yet fully understood^16^ ^,^ ^17^. As early as 1988, Demetriades et al.^18^ observed the possibility of spontaneous healing of the diaphragmatic muscle.

The literature is controversial and very poor when discussing the natural evolution of a small diaphragm injury^19^ ^-^ ^22^.

There are indications that, on the right side, these lesions may evolve to spontaneous healing. It is argued that in this situation the diaphragm finds a hepatic bed and that under certain conditions there is a possibility of spontaneous healing^23^ ^-^ ^26^. In fact, in a previous experimental study^27^ we found that in rats linear wounds of the right hemidiaphragm evolved with spontaneous healing.

Although wounds in the left hemidiaphragm do not have this favorable condition (presence of the liver) for healing, the question arise: is it possible that small linear wounds on the left side of the diaphragm also heal?

We idealized the current experimental study in rats, with the objective of clarifying the natural evolution of linear perforating wounds of 30% extension in the left diaphragm and the anatomopathological characteristics of their healing.

## METHODS

The project was approved by the Ethics Committee on Animal Use (CEUA) of the Irmandade da Santa Casa de Misericórdia de São Paulo, under protocol number 011/16, with opinions from the Departments of Surgery and Pathological Anatomy of the Irmandade, as well as from the Department of Physiology, Faculty of Medical Sciences, Santa Casa de Misericórdia de São Paulo. The work was accompanied by the team from the Animal Facility of the Department of Physiological Sciences. We used Wistar strains of Rattus norvegiccus albinus, submitted to a linear lesion of approximately 30% of the diaphragm through a regular and longitudinal incision in the muscle fibers of its left portion, between the lateral border of the diaphragm insertion to the thoracic lateral wall and the tendon center, without reaching them. We marked the lesions with 6.0 mononylon stitches at each end, to assist in its recognition at necropsy, for the removal of the specimen to be sent to histopathological study. The animals were about 90 days old (250g on average) and were kept in collective cages for 30 days, with a population density of five animals per cage, with free and unrestricted access to water and food recommended for rodents, in a twelve-hour light/dark cycle. The identification of each animal was made with a permanent ink marker in the tail and the ear. During the first 30 days of postoperative period, subjects were monitored for pain, calculated with the Grimace scale. After 30 days, the animals were submitted to necropsy with median laparotomy. The diaphragmatic fragments were sent for microscopy to evaluation of the type of healing.

**Figure 1:**
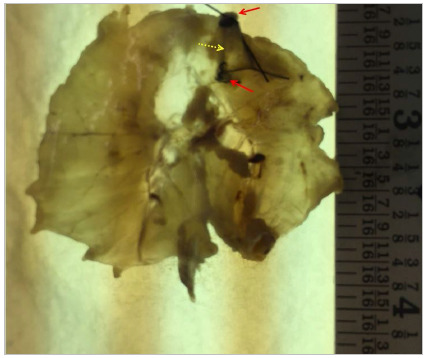
Diaphragm fixed in formalin, with two orientation points showing the ends of the surgical wound. Dotted arrow: Injury site; Solid arrows: orientation points.

The surgical specimens were removed and immersed in a flask with 10% formalin with approximately 10 times the volume of each fragment. They were submitted to macro and microscopic examination by the Pathology Service, with special stains for muscle and collagen, in addition to the evaluation of the presence of collagen. The macroscopic examination aimed to determine the size of the specimen and to visualize the lesion (whether healed or not), following the orientation points (Figure 1).

Finally, we performed sequential sections of the lesion and healthy regions. These sections were stored in small cassettes and returned to formalin. At this point, the histological preparation began. The sections were stained with hematoxylin-eosin, Masson trichrome and Picrossirius. At hematoxylin-eosin, collagen is pink and muscle fibers are red. In Masson’s trichrome stain, collagen is blue in color, while muscle tissue is red in color. And in the staining with Picrosirius, collagen presents with an intense red color and the musculature with a faint pink color. We wanted to clarify if we would only find fibrosis in the scar or if we would also have muscle fibers (hyperplasia).

We used semi-quantitative metrics (from the Institution’s own pathology laboratory) in the pathological evaluation:

Presence or absence of fibrosis:
  0: no fibrosis;
  1: focal fibrosis;
  2: more than one point of fibrosis;
  3: intercommunication between the fibrosis islands.
Presence or absence of muscle fibers:
  0: no muscle fibers;
  1: sparse muscle fibers;
  2: muscle fibers crossing the entire scar.

### Statistical analysis

To estimate the number of copies needed to carry out the study, we performed a sample size calculation based on the analysis of two previous studies of the same line of research, published in 2014 and 2015^27^ ^,^ ^31^. In these studies, diaphragmatic healing was observed in a percentage close to 50%. As per the work of Caiel et al.^31^, healing occurs between 40% and 63%, depending on the evaluation method. Therefore, considering the hypothesis that the percentage of healing varies between 35% and 65%, to obtain estimates with 95% confidence, the study required 43 animals. Assuming a loss of 10% of animals in the postoperative period, the minimum sample size was 48. We therefore decided to use 50 rats in this study.

Initially, we analyzed all variables descriptively. For the quantitative variables, we recorded the minimum and maximum values and computed the averages and standard deviations. For qualitative variables, we calculated absolute and relative frequencies. To compare the means of the two moments, we used the paired Student’s t test^28^. We evaluated the agreement between two methods using the Kappa index28 (for Kappa above 0.75 there is excellent reproducibility, between 0.40 and 0.75, good reproducibility, and below 0.4, marginal reproducibility). The software used for the calculations was the SPSS 17.0 for Windows. The significance level used for the tests was 5%.

## RESULTS

There were 50 rats operated. Mortality was 16% (8) in the immediate postoperative period: one due to anesthesia, two due to tetany after medication, and five due to respiratory complications in ventilation. Thus, 42 rats were alive at the end of the study.

We found only three diaphragmatic hernias and two cases without diaphragmatic healing, but without hernia, so the healing rate was 90%.

We evaluated 42 samples with lesions and prepared three slides for each sample with the respective stains: hematoxylin and eosin, Masson’s trichrome, and Picrosirius. Table 1 shows the description of fibrosis and muscle fibers at the lesion sites, with absolute and relative frequency of fibrosis in the injured areas. There was no statistical difference between the stains as for the observation of fibrosis. All of them were sensitive and specific to this finding. We did not observe interposed muscle fibers in the described fibrosis.

**Table 1:** Absolute and relative frequencies of fibrosis and muscle fibers.

Staining	Quantification of fibrosis	n	%
HE	0	7	16.7
	1	7	16.7
	2	9	21.4
	3	19	45.2
MASSON	0	5	10.0
	1	8	19.0
	2	9	21.4
	3	20	49.6
PICROSSIRIUS	0	5	10.0
	1	8	19.0
	2	8	19.0
	3	21	52.0
Quantification of Muscle Fibers			
Any staining	0	42	100

**Table 2:** Analysis of diaphragmatic healing according to Caiel et al, 2015. Macroscopic, Microscopic, and Tomographic Characterization of lesions.

Variable	Category	n	%
Macroscopy	Protection	23	57.5
	Hernia	17	42.5
Microscopy*	Protection	15	40.5
	Hernia	22	59.5
Variable	Category	n	%
Hernia diagnosed by tomography**	Protection	23	63.9
	Hernia	13	36.1
Total		40	100

*There are unknown values, as it was not possible to subsequently evaluate slides from three rats. **There are unknown values, as it was not possible to conclude the tomographic report of four rats.

**Figure 2:**
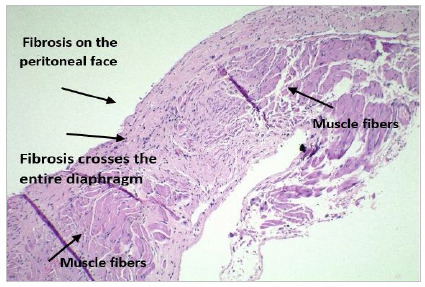
Hematoxylin-Eosin: Fibrosis crosses the entire lesion.

**Figura 3:**
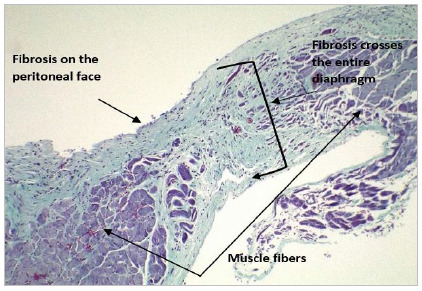
Masson trichrome: Fibrosis crosses the entire lesion.

**Figure 4:**
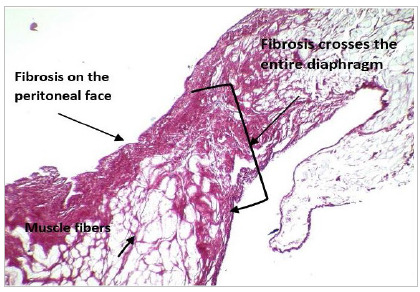
Picrossirius: Fibrosis crosses the entire lesion.

Following the analysis of the inflammatory response, there was fibrosis in different ways in the diaphragm, from an inflammatory process in the entire thickness of the diaphragm (“fibrosis crosses the entire lesion”), which occurred on two occasions (5%), to fibrosis on the peritoneal face in 28 (76%), and fibrosis affecting the peritoneal and pleural face in seven (19%) (Figures 2, 3 and 4).

In the histological analysis, we found only fibrosis causing scarring. Fibrosis was present In all cases.

## DISCUSSION

Initially, we deem worth mentioning that this work was experimental in rats, so the results obtained should be interpreted with caution and should not a priori be extrapolated to humans.

Like any experimental work, ours has limitations:

1. We analyzed only small and linear injuries resulting from puncture-cutting injuries.
2. We excluded complex diaphragmatic injuries or injuries resulting from blunt trauma.

When deciding to carry out the study with mice, the advantage of these animals was the low cost compared with other species, ease of accommodation, without the need for large spaces, and the characteristics of the diaphragm, which, despite being a very thin muscle, has great mobility during respiratory incursions and, consequently, supports an important pressure gradient, facilitating the procedure^29^.

Based on previous experiments^30^ ^,^ ^31^, we demarcated the lesion extremities with orientation points, so that it could be noticed in our macroscopic and microscopic evaluation.

The stains chosen were hematoxylin and eosin, Masson’s trichrome, and Picrossirius, because they have the power to differentiate collagen from muscle fibers and thus inform us if the scar contained, in addition to fibrosis, muscle tissue.

Healing occurred in 90% of animals. Fibrosis was present in all cases and the more specific the stain (Masson or Picrossirius) the greater the percentage of fibrosis found. There was no hyperplasia or muscle hypertrophy in the scar, only fibrosis. In addition, we observed fibrosis mainly on the abdominal side of the diaphragm, the pleural/thoracic face being spared from inflammatory reaction and fibrosis.

Although aware that the results obtained in experimental work with animals cannot be extrapolated to human beings, we understand that there is no other way to study biological phenomena other than in the laboratory. And so, it was done. In the laboratory, we analyzed whether small lesions of the left hemidiaphragm could heal, which we had already observed in the right hemidiaphragm^27^ ^,^ ^30^ ^,^ ^31^.

The main cause of diaphragmatic lesion is the injury to the thoracoabdominal transition zone, whose incidence varies from 20% to 48%^32^ ^,^ ^33^.

When the patient arrives at the emergency room with this type of injury, the question arises: is there a diaphragmatic lesion? We know that in up to 48% of cases there is an injury. When there are signs and symptoms that make diagnosis possible, such as presence of diaphragmatic hernia, imaging tests, etc., it is very easy to decide on the conduct. However, in approximately 10% of the cases, there is a diaphragmatic lesion and the patients are asymptomatic, mainly in cases of isolated lesions of the diaphragm. In this situation, how to make the diagnosis without invasive methods (videothoracoscopy)^34^? According to Demetriades et al.^18^, Madden et al.^5^, and Petrone et al.^21^, the diagnosis is unlikely without this exam, as there are currently no imaging methods that show it, and the patient will have late consequences in the future due to the presence of a complicated diaphragmatic hernia. Or could small diaphragmatic injuries such as those caused by stabbing or gunshot wounds heal spontaneously?

Authors such as Murray et al.^2^, Shaftan^35^, and Hegarty et al.^36^ claim that diaphragmatic wounds are not subject to spontaneous healing.

However, there are authors who, in selected situations, do not indicate operative treatment for thoracoabdominal injuries on the right side, believing in the healing of this muscle^23^ ^,^ ^37^.

What about left hemidiaphragm injuries? What’s their evolution^38^?

We were able to prove in this experiment that healing occurred in most cases of inflicted linear lesion of 30% of the surface of the left diaphragm, and that this healing is done through fibrosis only.

Furthermore, the best destination of a work, in addition to answering the question asked in the objective, is to raise a new doubt: in all the specimens studied, we observed the presence of fibrosis on the pleural surface, peritoneal surface and between these two layers. However, the fibrosis layer on the peritoneal surface was always thicker than on the pleural surface.

Why did this happen? We don’t know, we just observed it. It will be the subject of future study.

## CONCLUSION

Healing of the diaphragmatic lesion (30% linear) occurred on the left and it happened solely and exclusively by inflammatory process with collagen deposition.
